# Association between co-authorship network and scientific productivity and impact indicators in academic medical research centers: A case study in Iran

**DOI:** 10.1186/1478-4505-6-9

**Published:** 2008-09-16

**Authors:** Reza Yousefi-Nooraie, Marjan Akbari-Kamrani, Robert A Hanneman, Arash Etemadi

**Affiliations:** 1 Center for Academic and Health Policy, Tehran University of Medical Sciences, Tehran, Iran; 2 Students' Scientific Research Center, Tehran University of Medical Sciences, Tehran, Iran; 3 Department of Sociology, University of California, Riverside, USA; 4 Department of Epidemiology and Biostatistics, School of Public Health, Tehran University of Medical Sciences, Tehran, Iran

## Abstract

**Background:**

We aimed to examine the co-authorship networks in three successful Iranian academic research centers, in order to find the association between the scientific productivity and impact indicators with network features in a case study.

**Methods:**

We searched for English articles of the three research centers. We drew co-authorship maps of each center and calculated social network measures.

**Results:**

The collaboration networks in centers shared many structural features, including a "star-like" pattern of relations. Centers with more successful scientific profile showed denser and more cooperative networks. Key figures in each center were interviewed for their understandings of the reasons for the emergence of these patterns.

**Conclusion:**

Star shape network structure and dependency on a single big member is a common feature observed in our case study. Scientific output measures correlate with the network structure of research centers. Network analysis seems a useful method to explore the subtle scientific contexts in research organizations.

## Introduction

The process of research evaluation is of major importance for the development of health systems[1,2]. In this respect, centers within large medical schools are very important contexts for research production. Biomedical Research tends to be highly collaborative[3], and research institutions and academic centers bring researchers together in productive relationships. The quantity, quality, and creativity of the work produced in medical research centers vary. Much of the variation may well be due to the individual talent, expertise, and enterprise of the researchers. The structure of social relations, roles, and leadership, however, may also make critical differences.

Social network analysis provides a number of methods for revealing patterns of interpersonal relationships, and have frequently been applied to study collaboration through co-authorship networks [3-5]. Network analyses are becoming an important part in the growing body of research on social capital [6,7].

In the present study, we examined the co-authorship networks of researchers in three high profile Iranian research centers, and aimed to use it as a case study to investigate the association between the overall scientific productivity and impact indicators with collaboration network features.

## Methods

### • Co-authorship networks

Data were collected on co-authorship of papers published in English by members of three research centers: Endocrinology and Metabolism Research Center (EMRC), Digestive Diseases Research Center (DDRC) and Pharmaceutical Sciences Research Center (PSRC); which were the first three highest ranked research centers in Tehran University of Medical Sciences respectively and each has been at least once the top Iranian research center in the past 5 years [8]. These centers were selected because they had sufficient and similar number of articles to be analyzed. We searched the ISI Web of Science database for all articles, published since the establishment of each center until September 2006, referring to the center's name at the correspondence address. Because there was not any explicit membership definition for research centers, and there were a lot of people collaborating occasionally with the centers (in the form of theses or part time research), we defined the research center membership as having at least two articles affiliated by the corresponding center. In addition, the scientific secretaries of each center reviewed the lists to confirm the membership of selected authors.

We generated a table for each research center, with the rows formed by the list of members and the columns by the articles. A "co-authorship matrix" showing the number of collaborations between each pair of members was generated. The information regarding authors who were not considered as the members of centers was gathered as a single "outsiders" row.

### • Scientific productivity and impact indicators

We used ISI Web of Knowledge database to obtain following information for each article: the impact factors of the publishing journals (of the years of publication of the articles), and the number of citations received during two years after publication. We also obtained some other scientific productivity measures from an evaluation project conducted by the deputy for research of Tehran University of Medical Sciences in 2005–6[8]. These measures were the number of doctoral/master's theses, multi-centric research projects, obtained grants from external funding bodies, and seminars held by each center.

### • Analysis

The impact factors, the number of received citations and other scientific productivity measures were compared between three centers, and post hoc pairwise comparisons were performed when needed. Ucinet[9] and Pajek[10] software programs were used for network analysis of each center.

We calculated basic network descriptive measures and the average number of collaborators per author, regardless of the volume of production. To examine this, the data were dichotomized (i.e. if two actors co-authored any papers they were coded one) making a "collaboration matrix". Analyses of the structure of collaboration are based on the dichotomized data, showing whether two persons had ever collaborated.

Because of the considerable skewness of network measures, non-parametric measures of central tendency and dispersion were used. These measures were compared between the centers using Kruskall-Wallis test.

*Degree centralization*, and *betweenness centralization *measures were used to investigate whether collaboration is equally distributed across the researchers in each center, or there is a tendency for some actors to be more "central" to the web of collaboration [11]. At one extreme, a single individual or "star" may dominate – having far more collaborative connections than others, and acting as a broker or patron. At the other extreme, while there may be inequality, members are more able to form alternative collaborations, and are less likely to be dominated by the central elite. Star-shaped networks may be less productive than those with denser and more horizontal connections[12,13]. The *degree centralization *measure examines the inequality in the distribution of collaborators across researchers, varying between 0% to 100%, which shows the similarity of the network to star-shaped pattern[11]. The *betweenness centralization *measure examines the extent to which the shortest paths between pairs of actors in the collaboration network pass through a third actor [14]. To the extent that actors are "between" pairs of others, they may act as coordinators and brokers, and may gain status and power.

A frequent finding in collaboration networks is that they resemble "Small worlds"[15], particularly larger networks. That is, they have considerable clustering (*clustering coefficient*; which measures the probability that two of a scientist's coauthors have themselves coauthored a paper) combined with relatively low average *geodesic distances *(i.e. each actor in the network is "close" to all other actors). Creative scientific research on the one hand requires the close support of a community of peers who are expert in closely related areas, and on the other hand, needs getting access to the diverse sources of knowledge in other specialties. The simultaneous presence of dense local *clustering *with short social network *distance *to diverse others [15] is a characteristic feature of facilitated knowledge flow inside networks[16,17].

One approach to examine the structure of collaboration (social positions) is called "structural block modeling"[11]. In this approach, we group together actors who are similar in terms of the pattern of ties that they have to all other actors. The resulting simplified pattern shows who belong to which "cluster," and which clusters collaborate, or do not, with one another. We used a five or six block model to investigate the structural equivalence of the centers, determined by the overall goodness of fit.

Using different centralization measures, three most central actors were identified in each network. We interviewed some active members of each center and discussed the potential reasons for importance of core people, the similarities in the roles of members of clusters, and the relationships between the observed clustering and formal and informal categorizations in the centers.

## Results

### • Scientific productivity and impact indicators

The scientific activity measures of three centers are shown in table 1. The mean journal impact factor of DDRC was significantly more than two others (p: 0.0001). EMRC articles received the lowest citations among three centers (p: 0.003). The percentages of multi-centric projects and obtained grants from external bodies in PSRC were significantly less than other centers.

**Table 1:** Scientific productivity and impact indicators of three centers

	**DDRC**	**EMRC**	**PSRC**	**p-value**
Impact factor, mean (SD)	2.71(1.4)	1.37(0.99)	1.77(0.77)	0.0001
Received citations, median (interquartile range)	2(4)	0(1.25)	2(4.25)	0.003
Doctoral/master's theses (percentage of total running research projects)	30(43%)	17(33%)	7(30%)	0.4
Multi-centric projects (percentage of total running research projects)	32(46%)	18(35%)	1(4%)	0.001
obtained grants from external funding bodies(percentage of total running research projects)	18(26%)	12(24%)	1(4%)	0.09
seminars held	3	1	0	-

### • Co-authorship networks

Some descriptive statistics of three centers and their comparisons are shown in table 2. The mean papers per author and mean authors per paper in PSRC were significantly lower than two other centers.

**Table 2:** Bibliometric statistics and network measures in three centers

	DDRC	EMRC	PSRC	pvalue
Total papers	48	50	54	-
Total authors (size of network)	39	33	36	-
Papers per author (median(IQ range))	4(4)	4(4)	2(2.75)	P = 0.006
Maximum papers per author	42	43	22	-
Mean authors per paper	5(3)	4(2.2)	2.7(1.3)	p < 0.0001
Maximum authors per paper	12	11	6	-

**Co-authorship network**				

Average co-authorship (median(IQ range))	23(35)	18(15)	6.5(7.5)	p < 0.0001
Collaborators per author (median(IQ range))	14(9)	10(7.5)	5(3)	p < 0.0001

**Network centralization**				

Degree centralization	61.5%	63.8%	50.6%	-
Betweenness centralization	15.6%	27.7%	57.2%	-

**Small world phenomenon**				

Average density	.417	.369	.151	-
Clustering coefficient	.729	.717	.735	-
Mean geodesic distance	1.6	1.6	2.3	-

**Outsiders**				

Outsiders per author (median(IQ range))	21(26)	6(10)	5.5(5)	p < 0.0001
Spearman's correlation of inside and outside collaborations	0.8(p < 0.001)	0.49(p < 0.001)	0.32(p > 0.05)	-

#### Degree distribution

**Figure 1:**
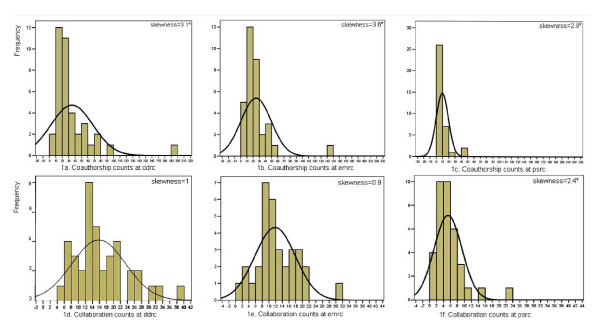
Co-authorship and collaboration count histograms in three centers with the associated normal curves and skewness measures.

There were notable differences in the absolute number of co-authorships between centers. All three centers displayed significant positive skewness in the distribution of co-authorship (figure 1). It was substantially greater in the DDRC and EMRC than in the PSRC. The overall productivity (median and mean papers per author) was also greatest in the former two centers.

The overall collaboration (regardless of the volume of production) in DDRC and EMRC was higher than PSRC; moreover, the distribution of the volume of production was more unequal than the distribution of collaboration.

#### Centralization

All three centers displayed high *degree centralization*, though it was less in PSRC. The DDRC and EMRC had a more characteristic single "star" pattern. The PSRC is characterized by two actors who were somewhat more involved in collaborations than the others, but similar to one another. The *betweenness centralization *was greatest at PSRC.

#### Small world phenomenon

As shown in table 2, all 3 centers displayed quite high levels of *clustering*. The average *distance *between members of the center, however, was much greater in PSRC (2.3) than DDRC or EMRC (both 1.6); This greater distance suggests that, in PSRC there were less actors who connect multiple clusters, showing a less typical "small-world".

#### Collaboration with outsiders

The DDRC was distinguished by the very high rate of collaboration with outsiders (non-members). The relative amount of collaboration "inside" and "outside" the organization was about the same for DDRC (14/21) and PSRC members (5/5.5) while EMRC members (10/6) showed a tendency toward relatively more "internal" activity.

Spearman's correlation coefficients for the association between the amount of inside and outside collaborations are shown in table 2. It was notably pronounced at DDRC which suggests it was the more central people who were most likely to have outside connections. This was notably less in PSRC, where outside ties are the same across all levels of the internal hierarchy.

#### Structurally equivalent groups

**Figure 2:**
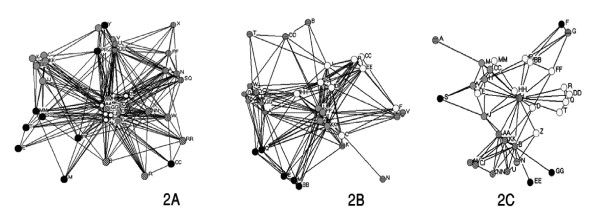
Collaboration network at DDRC (2A), EMRC (2B) and PSRC (2C) with structurally equivalent blocks colored.

The groups of "similar" actors, defined by *structural block modeling*, are shown with colors in figures 2A, B, and 2C. Data on central actors (defined by three centralization measures) in each center are shown in table 3.

**Table 3:** Central actors in each center based on three centrality measures

	Degree centrality		Betweenness centrality		Eigenvector centrality	
	Actor	Centrality	Actor	Centrality	Actor	Centrality

DDRC						
	AA	38	AA	118	AA	.289
	EE	32	EE	56	EE	.264
	J	30	J	38	J	.259
EMRC						
	FF	31	FF	143	FF	.334
	KK	21	J	22	KK	.273
	Z	20	A, KK	22	Z	.267
PSRC						
	L	22	L	351	L	.428
	B	14	B	129	B	.346
			AA	49	AA	.303

#### • DDRC

One plausible block model divided the actors into five groups (figure 2A). This model fitted the "ideal" pattern imperfectly (R-square = .425). Actors AA (head of the center), EE and J were defined as the most central people. We discussed the observed pattern of network with actors Z and HH. Both stated that, the three central actors played the most influential roles in promoting research projects and connecting the members. One reason for actor AA to be central was his position as the head of the center, and for actor EE as the head of the lab. Two interviewees considered actor J as central actor, mainly because of his personal abilities as a facilitator and promoter. The circles with vertical lines (the Cancer research team) are the current members of the oldest group of the center which many other groups stemmed from it. This group played an important role in internal and external communications of DDRC.

#### • EMRC

A five-group block model provided a plausible summary of the data for EMRC (R-square = .491). Structurally, the pattern of collaboration at EMRC was similar to that of DDRC. Compared with DDRC, local clusters were tighter, and more separated from one another (figure 2B). The role of the "core" in integrating the center was greater. Actors FF (head of the center), KK and Z were identified as most central. The observed network was discussed with actors JJ and J. Both stated that the three most central people possessed the key positions in the center (head, deputy and secretary respectively). Two most influential and relatively independent groups in EMRC were the Osteoporosis research team (mainly whites) and the Lipid research team (Vertical lines). FF, JJ and KK were the oldest members and founders of EMRC, and JJ is currently the head of the Osteoporosis research team. J is the head of Evidence based medicine research team, an independent entity working under EMRC's umbrella; therefore he showed high betweenness despite a non-central role.

#### • PSRC

A six group model was used, because one actor was unique in forming a "group" (figure 2C, circle with vertical lines). The overall fit of the model was moderate (R-squared = 0.456). There was a notable tendency towards thinner ties between some groups, suggesting a lower cohesion in PSRC.

There were two tight clusters (oblique lines and grey), which did hot have strong ties to one-another (figure 2C). Actors L (head of the center) and B were most central. The observed pattern was discussed with actors B and H. Both explained it mainly as a result of the existence of two distinct research disciplines of Clinical Pharmacology (oblique lines) and Medicinal Chemistry (grey and white) in the center, with different research methodologies and very rare common projects, and therefore infrequent relationships. Consequently, PSRC consisted of two star shaped independent networks. Actor B is the manager of the Clinical Pharmacology team.

## Discussion

The network structures associated with the scientific productivity and impact indicators in our case study. Centers with denser, more decentralized, and more open to outside connections networks showed better scientific outputs. Even though, all three centers shared many network features, demonstrating common infrastructural characteristics and obstacles of research organizations in developing countries.

All three networks were structurally star shaped and centralized. The central actors consisted of similarly the heads of the centers. This is a phenomenon which is seen in many team works in developing world. A single charismatic person, who initiates, maintains and pushes the organization. Two main reasons for this limited autonomy of members and involvement of the big person on almost all operations may be the lack of professional trained researchers who are able to manage the projects independently, and the instability of the system, in which only the organizations with support from a powerful actor are able to survive. This dependency could threat the sustainability of research centers and diminish the knowledge flow between members. Rulke and Galaskiewicz showed that hierarchical structures are not efficient for complicated problems while more intense and decentralized networks provide the maximal knowledge flow between actors [12]. In addition, the central actors in three centers possess other demanding responsibilities which reduce their ability to monitor all routines in the centers. The occurrence of such *betweenness *in few people in a network makes it highly susceptible to break down after the removal of such actors[18].

One explanation for the observed difference in the average authors per paper in three centers is the higher likelihood of guest authorship, because of the presence of more prestigious and socially important members in some centers. Guest authorship is prevalent in scientific papers[19]. As showed by Bhopal et al. in many situations it happens unintentionally, and the powerful people may be unaware of being included as author by their novice peers[20]. In many Iranian research centers research is performed by novice general practitioners or clinical faculty members who work as part time amateur researchers. This lack of professionalism may enhance such phenomenon.

In our case study one network (DDRC) showed denser and more distributed network measures (highest average co-authorship, average collaboration, *density*, and least *betweenness centralization*, and *mean geodesic distance*). The scientific output measures were consistent with the network findings; depicting that the production of more cited research (a surrogate of higher quality research) seems to be associated with the inter-actor associations and knowledge flow inside research organizations.

DDRC members had more collaboration with outsiders, which made the center more open, and facilitated the potential of idea exchange and conducting interdisciplinary and multi-centric projects. Research centers are communities of a sort. At one extreme, they may be very "open" where members are equally likely to have ties outside, as inside the center. At the other extreme, they may be quite "closed". Some degree of closure probably contributes to "identity" and a "sense of mission". Too much closure may contribute to isolation from the larger world of science. It also may matter where the openness occurs. In many centers, the central figures and leaders act as the primary liaison between the center and the larger community; in other centers, many individuals – and not always central individuals – may have strong external ties. The former pattern may contribute to *cohesion *and *collaboration*; the latter pattern may contribute to *factionalization *and lower *social density*, which was seen more apparently in PSRC network, where articles received relatively high citations while network showed lowest productivity measures comparing to other centers. One reason for PSRC's high median citations per article in spite of lower productivity indicators and network measures, is the attractiveness of pharmacological research in comparison to clinical research, as shown by other scientometric studies [21].

An archetypical small-world network as stated by Moody "will have many distinct clusters, connected to each other by a small number of links" [22]. This *clustering *preserves the independence and autonomy of the clusters, while the paths of the knowledge exchange among them are open. DDRC and EMRC showed to be more typical small worlds. Several studies have demonstrated that the information diffusion and knowledge exchange are faster in small worlds[15,23]. Coexistence of dense and weak relationships in small worlds showed to enhance innovation. Because the dense and clustered relationships raise trust and cooperation, while ties to other clusters bring new information to the cluster[24,25].

PSRC had lowest mean *authors per paper*, mean *papers per author*, *density*, and highest mean *geodesic distance *between members, and *betweenness centralization*. Lower mean authors per paper means the involvement of smaller number of researchers in common projects, and lower potential for team working. This measure highly depends on the research discipline. Newman showed that the average authors per paper is 3.75 in biological sciences, 2.53 in physics and 1.45 in mathematics. He explained this difference because of involvement of large groups of field and laboratory scientists in biological research in comparison to more theoretical and individual nature of mathematical sciences [26].

Taking a closer look at networks and inter-relationships between entities as an important aspect of sustainable capacity development [27] is essential for developing countries. This warrants the assessment and development of interpersonal and inter-institutional networks to set up durable enhancing frameworks for innovation [28]. Network analysis methods seem effective and meaningful.

Our study is limited in some ways. Co-authorship relationship seems a rational- but stringent- definition of scientific collaboration. Knowing each other is a pre-requirement for writing a paper together [3]. Co-authorship networks do not absolutely reflect the scientific collaboration, because many researchers may know and influence each other but never collaborate in writing a paper. In addition, several factors may interact on co-authorship events; many of which are not purely scientific. We limited our study to the published English language articles containing the center name in the ISI address field. This approach omits the Persian language articles and those that are written by the members but do not include the center's name in the address field. We also used a tough method for defining the membership. This may overestimate the number of outsiders, because of the presence of part time members of research centers who were considered outsiders in our study.

The scientific productivity and impact of research organizations was associated with their network structure in our case study. Social network analysis seems a useful method to explore and visualize the subtle scientific norms and customs in research centers and hidden structural and functional sources of their success or failure. Qualitative approaches to obtain the network members' views regarding network analysis outputs lead in more meaningful and less mechanical interpretations of the hidden structure.

## Competing interests

The authors are not members of any of the research centers under study. No conflicts of interests are declared. AE is working as an associate editor in a peer reviewed journal which is founded by the dean of one research center under study.

## Authors' contributions

conception of the research: RYN, MAK, AE; performing the research: RYN, MAK; data analysis and interpretation of findings: RYN, MAK, RAH; preparation and reviewing of manuscript: RYN, MAK, RAH, AE

## References

[B1] Groneberg-Kloft B, Scutaru C, Kreiter C, Kolzow S, Fischer A, Quarcoo D (2008). Institutional operating figures in basic and applied sciences: Scientometric analysis of quantitative output benchmarking. Health Res Policy Syst.

[B2] Hanney S, Gonzalez Block M (2008). Why national health research systems matter. Health Res Policy Syst.

[B3] Newman M (2001). The structure of scientific collaboration networks. Proceedings of the National Academy of Sciences of the United States of America.

[B4] Otte E, Rousseau R (2002). Social network analysis: a powerful strategy, also for the information sciences. Journal of Information Science.

[B5] Kretschmer H, Aguillo I (2004). Visibility of collaboration on the Web. Scientometrics.

[B6] Burt R (1997). The contingent value of social capital. Administrative Science Quarterly.

[B7] Scott C, Hofmeyer A (2007). Networks and social capital: a relational approach to primary healthcare reform. Health Res Policy Syst.

[B8] Samadi N, Alhani Z, Jadidi M, Kharaman Z (2006). Evaluation of research activities of research centers of Tehran University of Medical Sciences (2005–2006).

[B9] Borgatti S, Everett M, Freeman L (2002). Ucinet for Windows: Software for Social Network Analysis. [6].

[B10] Batagelj V, Mrvar A (2000). Pajek program for large network analysis. [1.02].

[B11] Hanneman R, Riddle M (2005). Introduction to social network methods.

[B12] Rulke D, Galaskiewicz J (2000). Distribution of Knowledge, Group Network Structure, and Group Performance. Management Science.

[B13] Back K (1974). Intervention techniques: Small groups. Ann Rev Psych.

[B14] Hawe P, Webster C, Shiell A (2004). A glossary of terms for navigating the field of social network analysis. J Epidemiol Community Health.

[B15] Watts D (1999). Small Worlds: The dynamics between order and randomness.

[B16] Watts D, Strogatz S (1998). Collective dynamics of small world networks. Nature.

[B17] Flemming L, King C, Juda L (2004). Small Worlds and Innovation.

[B18] Holme P, Kim BJ, Yoon CN, Han SK (2002). Attack vulnerability of complex networks. Phys Rev E.

[B19] Bennett D, Taylor D (2003). Unethical practices in authorship of scientific papers. Emergency Medicine.

[B20] Bhopal R, Rankin J, McColl E, Thomas L, Kaner E, Stacy R (1997). The vexed question of authorship: views of researchers in a British medical faculty. BMJ.

[B21] Adams J, Gurney K, Marshal S (2007). Profiling citation impact: A new methodology. Scientometrics.

[B22] Moody J (2004). The Structure of a Social Science Collaboration Network: Disciplinary Cohesion from 1963 to 1999. American Sociological Review.

[B23] Yamaguchi K (2002). The structural and behavioral characteristics of the smallest-world phenomenon: minimum distance networks. Social Networks.

[B24] Cowan R, Jonard N (2003). The dynamics of collective invention. Journal of Economic Behavior and Organization.

[B25] Cowan R, Jonard N (2004). Network structure and the diffusion of knowledge. Journal of Economics Dynamics and Control.

[B26] Newman M (2004). Coauthorship networks and patterns of scientific collaboration. Proceedings of the National Academy of Sciences.

[B27] UNDP (2004). Scientific Capacity in Developing Countries, Postnote, No 216, March.

[B28] Cooke J (2005). A framework to evaluate research capacity building in health care. BMC Fam Pract 2005.

